# Decomposing sensorimotor variability changes in ageing and their connection to falls in older people

**DOI:** 10.1038/s41598-018-32648-z

**Published:** 2018-09-28

**Authors:** Chin-Hsuan Lin, A. Aldo Faisal

**Affiliations:** 1 Brain & Behaviour Lab, Department of Bioengineering, Imperial College London, South Kensington Campus, SW7 2AZ London, UK; 2 Department of Computing, Imperial College London, South Kensington Campus, SW7 2AZ London, UK; 3 MRC London Institute of Medical Sciences, W12 0NN London, UK

## Abstract

The relationship between sensorimotor variability and falls in older people has not been well investigated. We developed a novel task having shared biomechanics of obstacle negotiation to quantify sensorimotor variability related to locomotion across age. We found that sensorimotor variability in foot placement increases continuously with age. We then applied sensory psychophysics to pinpoint the visual and somatosensory systems associated with sensorimotor variability. We showed increased sensory variability, specifically increased proprioceptive variability, the vital cause of more variable foot placement in older people (greater than 65 years). Notably, older participants relied more on the vision to judge their own foot’s height compared to the young, suggesting a shift in multisensory integration strategy to compensate for degenerated proprioception. We further modelled the probability of tripping-over based on the relationship between sensorimotor variability and age and found a correspondence between model prediction and community-based data. We reveal increased sensorimotor variability, modulated by sensation precision, a potentially vital mechanism of raised tripping-over and thus fall events in older people. Analysis of sensorimotor variability and its specific components may have the utility of fall risk and rehabilitation target evaluation.

## Introduction

Falls are the leading cause of unintentional injuries in older people^1,2^; they account for more than 60% unintentional injuries and 50% of accidental death among people aged 65 and older^1^. Most falls in older people occur during locomotion, and more than 50% of falls are triggered by tripping over something during walking^3–7^. Biomechanical research has documented gait differences between the young and older that are related to falls. Among them, gait variability has been consistently observed to increase with age^8–10^, and older fallers have higher variability than older non-fallers^8^. Increased gait variability is thus considered a marker of increased risk of falling. Although these epidemiological and biomechanical studies have provided phenomenological understandings of falls, the physiological basis underlying older people’s gait variability changes remains largely unexplored.

Walking, like other motor tasks, is achieved by making use of sensory information, which conveys the states of the environment and body, to generate and execute motor commands^11–13^. The neural signals, from sensation, motor planning to execution, are intrinsically noisy^11,14^. This noise in the sensory and motor systems causes variations of motor performance across multiple repetitions of a task, i.e. sensorimotor variability^13–15^. Most studies probing the ageing effects on sensorimotor tasks have focused on the upper limb and consistently found higher sensorimotor variability in older people^16–19^. This increase of sensorimotor variability is associated with a deficiency in older people’s activities of daily living^16,20^. A small number of studies compared ankle positioning precision between the young and older^21,22^ and showed increased position variability in older people. It is thus reasonable to hypothesise that increased sensorimotor variability with age also presents in locomotion and manifests as increased gait variability. Further, this raised sensorimotor variability in the lower limb would impact negatively on older people’s health and quality of life by increasing their fall risk.

Evidence has supported that ageing-related deficits of multiple sensorimotor functions are linked to gait decline and an increased probability of falling^23–27^. However, the majority of these studies focused only on average performance metrics, but not variability. In the small number of studies investigating variability values, one study showed a link between greater quadriceps muscle unsteadiness and fall history in older people^25^. Regarding sensation, the roles played by vision and proprioception in falls have been investigated mainly through balance control. For example, studies have revealed that raised ankle joint position sensation variability during ageing are likely to contribute to impaired static postural control in older people^28,29^. Much less is known about the association between sensory degeneration and sensorimotor variability during ageing, let alone gait variability. We argue that there is a need to delineate the connection between sensory variability and gait impairments with age because this approach links sensory degradation to trip-over risk when walking.

There is increasing evidence showing that people make use of visual estimates of the environment (see review^30^) as well as visual^30^ and proprioceptive estimates of the extremity position^31^ to guide their locomotion. The gross sensory variability of walking thus originates from the visual uncertainty of the environment as well as the visual and proprioceptive uncertainty of the extremity. Vision^23,32^ and proprioception^31,33^ are susceptible to ageing process, but the scales and speed of changes may differ. To date, there has been no systemic investigation to pinpoint the relevance of the variability of individual sensory modalities to ageing-related sensorimotor variability alterations. As modality specific measurements can lead to a more precise understanding of mechanisms behind increased sensorimotor and gait variability and thus the development of targeted fall prevention strategies, it is valuable to evaluate visual and proprioceptive variability separately.

Of many gait variables, minimum foot clearance (MFC), the minimum vertical distance between the lowest surface of the foot/shoe and the ground surface during the mid-swing phase of gait, is considered to be directly linked to trip-over occurrence^8,10,34,35^. This is because that a foot or obstacle-ground encounter (i.e. a trip-over) occurs when MFC equals to or is lower than zero^8,10^. Extensive investigations during the past two decades have identified greater MFC variability in older people, especially older fallers in multiple studies and regarded it as an important risk factor for falls^8,10,34,35^.

Taking the evidence as mentioned above together, we hypothesised that increased sensorimotor variability with age manifests as increased MFC variability and thus increased trip-overs and falls. Moreover, raised sensory variability due to degeneration contributes to increased sensorimotor variability. The objective of this study is thus to characterise sensorimotor and sensory variability in the context of MFC. Besides, we aim to quantify the visual variability of the environment as well as the visual and proprioceptive variability of the extremity. To do so, we first develop a controlled motor paradigm biomechanically mimicking stepping, FOot HEight POsitioning (FOHEPO) task, to compare sensorimotor variability in different age groups. We then use sensory psychophysics to measure gross sensory variability and the sensory variability of the environment, both in the context of the FOHEPO task. Because the visual and proprioceptive variability of the extremity is not directly measurable, we employ two well-established computational principles in human perception to estimate it. First, it is known that overall sensory variability is the sum of variability originating in the environment and body^36,37^. Thus, by subtracting environment sensory variability from gross sensory variability, extremity sensory variability can be acquired. Second, the Bayesian integration rule^11,38,39^ states how combining visual and proprioceptive information of limb positions is based on separate variability. We can, therefore, estimate the proprioceptive and visual variability of the extremity by manipulating the visibility of the extremity and comparing differences between limb visible/invisible conditions. We additionally perform predictive modelling to indicate the probability of tripping-over while negotiating structured obstacles (i.e. staircases) using sensorimotor variability at different ages.

## Methods

**Figure 1:**
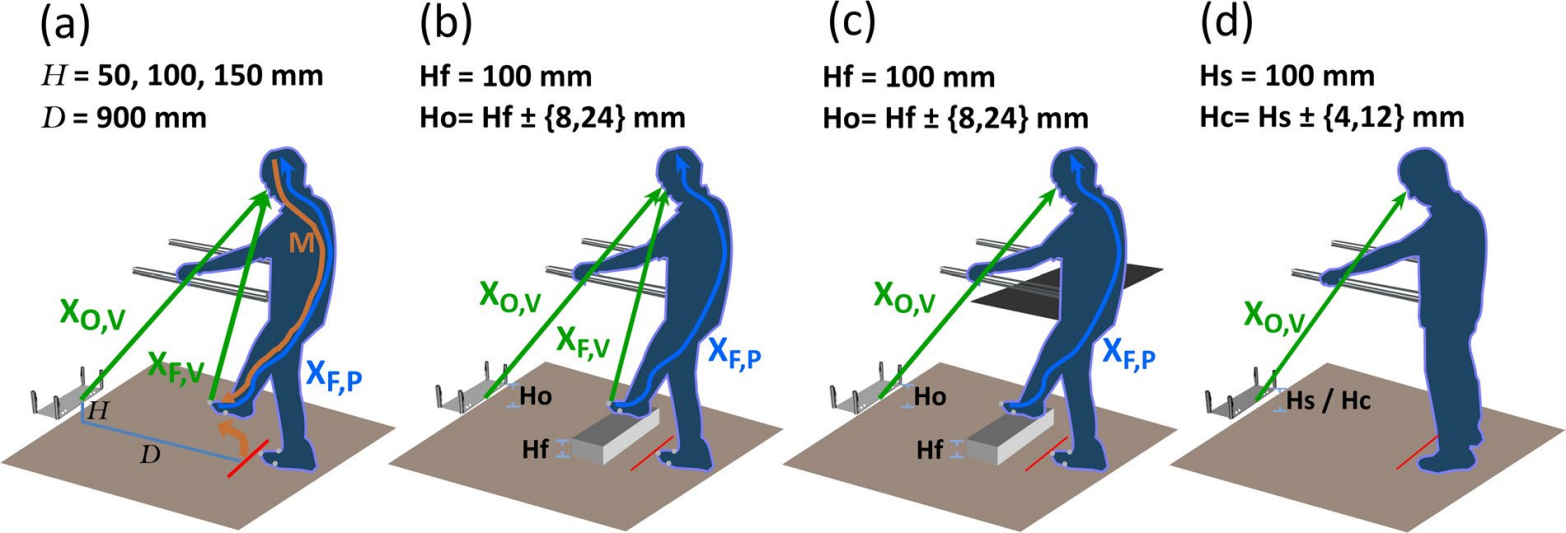
Experimental set-up. Participants stood inside an automated robotic platform, 900 mm away from the obstacle. They undertook four tasks. In **(a)** FOot HEight POsitioning (FOHEPO) task, participants lifted one of their feet to match the height *H* of the obstacle. Three target heights were examined (50 100 and 150 mm). The FOHEPO task measured sensorimotor variability. Sensory variability in the context of the FOHEPO task was then measured by using **(b)** Foot-obstacle two-alternative forced choice (2AFC), leg-visible condition. In each trial, one of the participant’s feet was lifted by a 100 mm high platform (**Hf** 100 mm). The obstacle height **Ho** was randomly chosen from 4 levels: 100−24, 100−8, 100 + 8 and 100 + 24 mm. Participants chose whether the lifted foot or the obstacle is higher in each trial. We then conducted two more sensory psychophysics **(c)** and **(d)** to compute sensory variability originated from individual sensory modalities, including vision and proprioception. In **(c)** Foot-obstacle 2AFC, leg-invisible condition. The procedure was identical to **(b)**, except that the visual information of foot height was blocked by a piece of A1 size black paper. **(d)** Obstacle 2AFC. Each trial consisted of two successive observation intervals. The standard stimulus (**Hs** 100 mm) randomly appeared in one of the two intervals. The comparison stimulus **Hc** was randomly chosen from 4 levels: 100−12, 100−4, 100 + 4 and 100 + 12 mm. Each interval presented the stimulus for 2 seconds. Between these two intervals, actuators moved the obstacle to the position of the second interval. Participants then chose the interval containing the higher height between the two at the end of each trial.

Four experiments were conducted (Fig. 1). In experiment 1, the FOot HEight POsitioning (FOHEPO) task, sensorimotor variability of foot stepping was measured. Experiment 2, 3 and 4 were classical constant stimulus sensory psychophysics with two alternative force choice (2AFC) tasks. In experiment 2, height discrimination between the foot and obstacle was used to quantify the sensory variability of the FOHEPO task. The procedure of experiment 3 was identical to experiment 2 except that the visibility of the foot, leg and thus the height was blocked. We compared the sensory variability in experiment 3 to the variability in experiment 2 to analyse the visual occlusion impact. Experiment 4 was obstacle height discrimination. Taking together values acquired in experiment 2, 3 & 4, we computed the variability of individual sensory modalities. We then built a probability model to predict fall occurrence as a function of age based on the data from experiment 1.

### Participants

Seventy-four people, including 30 young (13 females; mean 27.0 (S.D. 3.9) years, pre-defined range 20–40 years), 26 older (11 females; mean 73.3 (S.D. 4.4) years, pre-defined range 66 years and beyond) and 18 middle-aged (10 females; mean 59.5 (S.D. 3.9) years, pre-defined range 45–65 years) volunteered in the FOHEPO task. Young participants were graduates of Imperial College. Older and middle-aged participants were volunteers from the local community or Imperial College. The study was approved by the Imperial College Research Ethics Committee and performed in accordance with the declaration of Helsinki. All participants gave written informed consent to participate in the experiment. Participants completed a health questionnaire to ensure they had no past or present conditions that could interfere with the sensorimotor control and Waterloo Footedness Questionnaire-Revised (WFQ-R)^40^ to determine their dominant foot. All but one young participant were right dominant. Out of 74 who completed the FOHEPO task, 17 young (9 females, mean age 27.5 (S.D. 4.0) years), 22 older (11 females, mean age 69.8 (S.D. 5.8) years) and 17 middle-aged (9 females, mean age 59.5 (S.D. 3.9) years) volunteers completed the foot-obstacle 2AFC (experiment 2 & 3). Eighteen young (10 females; mean 28.6 (S.D. 4.7) years), 23 older (11 female, mean age 69.8 (S.D. 5.9) years) and 17 middle-aged (9 female, mean age 59.5 (S.D. 3.9) years) completed the obstacle 2AFC task (experiment 4). All the participants who did not complete all tasks exited the study due to relocation, except that one older participant withdrew after completing the FOHEPO task and foot-obstacle leg visible 2AFC because of an exercise injury, and one middle-aged participant lost contact. Data were unusable in one of the FOHEPO task (in the young group) and one of the foot-obstacle 2AFC (in older people group). There were no significant group differences in mean height or weight or gender distribution.

### Stimuli and apparatus

All experiments were performed in the FOHEPO workstation, a closed-loop robotic environment we built for high precision motor and sensory psychophysics on the lower extremities^41^. The workstation constituted of a supportive metal frame with its rear side open for a participant standing inside the workstation. The stimulus (We called it the ‘obstacle’ because it served as an obstacle that a subject had to step to.), a white polystyrene sheet L 760 × W 300 × H 5 mm was located at the front side of the workstation and surrounded by the black workstation walls and floor. Four Firgelli linear actuators (Firgelli L16-140-63-12-S, Firgelli, Victoria(BC/Canada) drove the obstacle moving vertically from 0 (=ground level) to 150 mm. Three Optitrack Flex-13 cameras (Natural Point, OR, USA) tracked the positions of participants’ feet and the obstacle at 120 Hz, using markers placed on the front of feet and the obstacle.

### Procedure

#### Experiment 1: FOHEPO task

(Figure 1a) Participants stood 900 mm from the obstacle and matched their foot to three target heights (50, 100, and 150 mm) while holding safety handrails. Twenty trials were presented for each target in each foot in a pseudorandom order. Each trial started with actuators moving the obstacle to the target height. Five hundred ms after the obstacle reaching its target level, a countdown phase with an audio signal being’three-two-one-beep’ was delivered. Participants got prepared while hearing the countdown audio but only started moving the designated foot on the beep. They flexed their knee and hip to raise the foot and kept the moving foot parallel to the ground. When considering their foot matching the obstacle height, participants pressed a button on the left handrail to transmit a time monitoring flag to the PC-controlled MATLAB Module. They kept their foot at the same level for 1000 ms until another audio signal, which indicated the end of a trial and provided performance feedback using real-time height difference acquired from motion tracking data (positive feedback when the difference between the foot and the obstacle less than 35 mm; otherwise negative feedback), played. If a participant did not press the button within 4000 ms, the trial was terminated automatically and labelled as “mistrial”. Participants were informed before the experiment that they would have one more chance to repeat mistrials at the end of each block. They also knew that the speed of reaction was not the primary measure and they could spend as much time on a trial provided that they complete it within 4000 ms. İf a participant failed to complete a trial in the second try, that trial was excluded from data analysis.

#### Experiment 2 and 3: Foot-obstacle 2AFC – leg visible and invisible conditions

(Figure 1b,c) Each condition contained 10 blocks of 12 trials with both feet being tested. A trial consisted of simultaneous presentations of one of the participant’s feet, which was lifted to 100 mm above the ground by a platform, and the obstacle. Foot heights were taken as standard stimuli. The obstacle heights, varying from ±8 and ±24 mm to the standard were presented as comparison stimuli. Participants chose the higher stimuli by pressing buttons on a numeric keyboard, on which button “F” meant “foot” higher and button “O” meant “obstacle” higher. Two conditions, one with the lifted leg visible and the other with the leg visually blocked, took place on two separate days. The orders of conditions and feet were counterbalanced among participants.

#### Experiment 4: Obstacle 2AFC

(Figure 1d) The experiment consisted of 10 blocks of 12 trials. Each trial comprised the sequential manifestation of obstacle stimuli. The height of the standard was 100 mm. The comparison was a set of heights from ±4 and ±12 mm to the standard. Between trials and intervals, linear actuators moved the obstacle. Participants closed their eyes between intervals and trials. Sound signals indicating the beginning and end of an interval were played to instruct participants when to observe the height of the obstacle front border. At the end of a trial, participants chose the higher interval by pressing buttons on a numeric keypad, on which button “1” represented the first interval and button “2” represented the second.

### Data processing and statistical analysis

Data analysis was performed by MATLAB (Release 2014a, Mathworks, Inc. Natick, MA, USA). In the FOHEPO task, the average of 1 second (120 frames) front marker height data, beginning from button press time, was regarded to be foot height, so as the height of the obstacle. Statistical analysis was performed by SPSS (IBM SPSS Statistics for Windows, Version 22.0&25.0. Armonk, NY: IBM Corp.). The Kolmogorov-Smirnoff test was used to check the normality of the data. For normally distributed data sets, we used mixed-design ANOVAs with post-hoc t-tests corrected for multiple comparisons with the Bonferroni method to provide control of the family-wise error rate^42^. Traditional frequentist testing does not provide a quantitative measure of how firmly the data support the null hypothesis. Therefore, for cases in which ANOVAs did not display significance, we conducted Bayes Factor (BF) analyses of independent sample t-tests for each pair to quantify how likely the null or alternative hypothesis was^43^. For data sets that were not normally distributed, the nonparametric Kruskal-Wallis H test was used. Outliers were detected by Tukey’s method^44^, taking (Quartile3) + (3 IQR) as a cut-off value.

#### Fitting psychometric functions and computing sensory variability

We used psychometric function parameters to estimate variability in experiment 2, 3, and 4. The computation procedures were as follows:

Raw responses Ψ were the proportion of trials in which a comparison stimulus was judged higher than a standard at each comparison level. We fitted raw responses to cumulative Gaussian distribution functions^45^.

1 $$\Psi =\lambda +\mathrm{(1}-2\lambda )\cdot {\mathscr{N}}(\alpha ,{\sigma }^{2})$$

By fitting the eq. (1), we obtained *σ*^2^, the variance of sensory estimate distribution of each experiment^36,46^. Other terms in the equation were *α*, the comparison height corresponding to the 50% point of the function and *λ*, the lapse rate.

**Figure 2:**
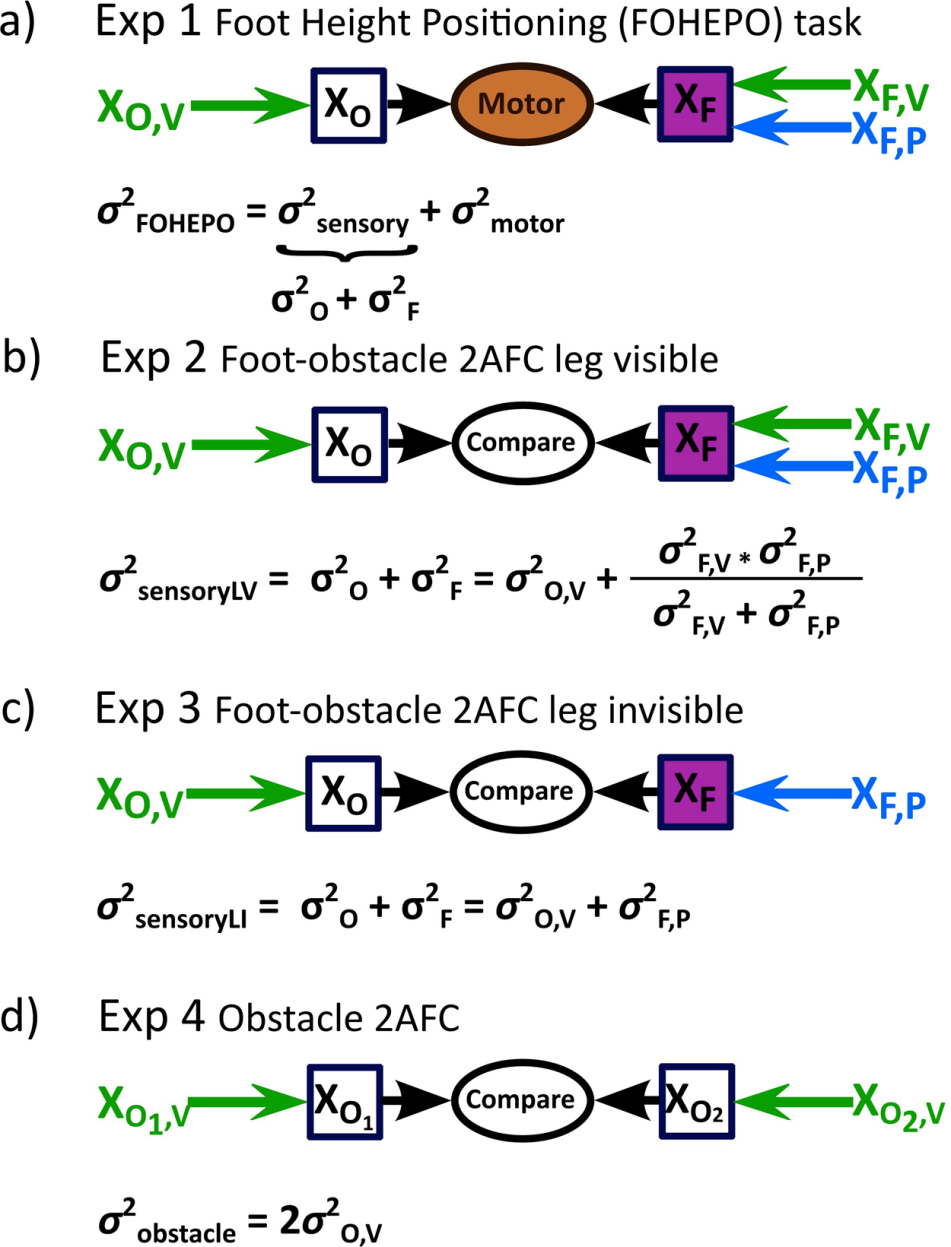
Models to represent and compute the variability of each task **(a)** In the FOHEPO task, task variability, i.e. sensorimotor variability, is the sum of sensory and motor variability [61]. The motor policy formed for the FOHEPO task is based on all available sensory information, including sensory estimates of the height of the obstacles (*X*_*O*_) and sensory estimates of the height of the own foot (*X*_*F*_). Therefore, the sensory variability of the FOHEPO task is the sum of obstacle height estimate variability ${\sigma }_{O}^{2}$ and foot height estimate variability ${\sigma }_{F}^{2}$^36^. **(b)** We directly measured the sensory variability using the foot-obstacle 2AFC leg-visible condition. As has been mentioned, the sensory variability, expressed as the variance of encoded position estimates of foot and obstacle ${\sigma }_{SensoryLV}^{2}$, is the sum of of the variances of obstacle ${\sigma }_{O}^{2}$ and the foot height estimates ${\sigma }_{F}^{2}$. Sensory estimation of the obstacle height is represented only by the visual information. Therefore, the sensory variability of obstacle height (${\sigma }_{O}^{2}$) equals to the visual variability of the obstacle height (${\sigma }_{O,V}^{2}$). Sensory estimates of foot height in the leg-visible condition are formed by combining the variance of proprioception $({\sigma }_{F,P}^{2}$) and vision (${\sigma }_{F,V}^{2}$) in a Bayesian way^11,38^. **(c)** When the lifted leg is invisible, foot height is estimated only based on proprioceptive representation and thus ${\sigma }_{F}^{2}$ equals to ${\sigma }_{F,P}^{2}$. Total sensory variance $sigm{a}_{SensoryLI}^{2}$ is still the linear sum of ${\sigma }_{O}^{2}$ and ${\sigma }_{F}^{2}$. **(d)** In the obstacle 2AFC, the visual estimates of obstacle positions at two intervals of a trial are compared. We assumed the variances of obstacle height estimates remain constant in two intervals. From the signal detection theory of psychophysics^46^, we know ${\sigma }_{obstacle}^{2}=2\cdot {\sigma }_{O,V}^{2}$, where ${\sigma }_{obstacle}^{2}$ is attainable from the psychometric function of obstacle 2AFC responses.

In experiment 2 and 3, total variability *σ*^2^ was the sum of obstacle height estimate variability ${\sigma }_{O}^{2}$ and foot height estimate variability ${\sigma }_{F}^{2}$ and can be expressed as the following eqs (2) and (3) (see also Fig. 2b,c):

Experiment 2: foot-obstacle 2AFC – leg-visible(LV)

2 $${\sigma }_{SensoryLV}^{2}={\sigma }_{O}^{2}+{\sigma }_{F}^{2}={\sigma }_{O,V}^{2}+\frac{{\sigma }_{F,V}^{2}\cdot {\sigma }_{F,P}^{2}}{{\sigma }_{F,V}^{2}+{\sigma }_{F,P}^{2}}$$

Experiment 3: foot-obstacle 2AFC – leg-invisible(LI):

3 $${\sigma }_{SensoryLI}^{2}={\sigma }_{O}^{2}+{\sigma }_{F}^{2}={\sigma }_{O,V}^{2}+{\sigma }_{F,P}^{2}$$

${\sigma }_{SensoryLV}^{2}$ represented the variance of the difference distribution and also the sensory variability ${\sigma }_{sensory}^{2}$ of the FOHEPO task. The height of the obstacle was only represented by vision and thus ${\sigma }_{O}^{2}$ equalled to the visual variability of the obstacle height ${\sigma }_{O,V}^{2}$. The variance of foot estimates in experiment 2 was a product of visual and proprioceptive variances of foot estimates. The product equalled $\frac{{\sigma }_{F,V}^{2}\cdot {\sigma }_{F,P}^{2}}{{\sigma }_{F,V}^{2}+{\sigma }_{F,P}^{2}}$ because we assumed that foot height estimation follows the Bayesian integration rule^11,38^, as previously modelled. This means different sensory modalities representing the same events being integrated in a fashion such that the reciprocal of the squared standard deviation (variance) of multi-sensory representation equals the sum of the reciprocals of uni-modal variances. In this way, the brain weights evidence based on the reliability of each sensory modality and minimises the variance in the final estimate. In the eq. (3), with only proprioceptive information of foot positions available, ${\sigma }_{F}^{2}$ equalled to ${\sigma }_{F,P}^{2}$.

In experiment 4 (see also Fig. 2d), there was such a relationship between the measured ${\sigma }_{obstacle}^{2}$ and the variance of obstacle estimates ${\sigma }_{O}^{2}$ based on literature^46^:

4 $${\sigma }_{obstacle}^{2}=2\cdot {\sigma }_{O}^{2}=2\cdot {\sigma }_{O,V}^{2}$$

Therefore, ${\sigma }_{O,V}^{2}$ equalled $\frac{{\sigma }_{obstacle}^{2}}{2}$. With ${\sigma }_{O,V}^{2}$ now being known, we could acquire ${\sigma }_{F,P}^{2}$ by subtracting ${\sigma }_{O,V}^{2}$ from ${\sigma }_{SensoryLI}^{2}$ (based on eq. (3)). Last, since ${\sigma }_{SensoryLV}^{2}$ and ${\sigma }_{O,V}^{2}$ are measurable and ${\sigma }_{F,P}^{2}$ could be deduced, we could compute ${\sigma }_{F,V}^{2}$ using eq. (2).

#### Modelling tripping-over occurrence

We related yearly tripping-over occurrence as a function of age to population data on falls during stair negotiation. To link falls per year to the trip-over probability per staircase we set up the following inference pipeline.

1. Trip probability per-step prediction: We built a model predicting the trip-over probability per step taken on negotiating structured obstacles, specifically ascending stairs with 150 mm risers. We fitted the sensorimotor variability data of the 150 mm condition of the FOHEPO task to a linear model which predicted the variability as a function of age
  5 $${\sigma }_{150}=11.06+0.09\cdot age$$
  We assumed foot clearance data during stair climbing distribute normally based on literature^47^ and our own data (Supplementary Material Fig. 4). We used published foot clearance to low-rise staircases (as our 150 mm example case) from a study by Riener and colleagues^48^ as the mean *μ* and standard deviation σ_150_ of our modelled foot distributions. We then computed the per-step probability of tripping-over *p*_*trip*_, i.e. the cumulative probability of foot placement lower than a 150 mm high staircase, using the following equation
  6 $${p}_{trip}={\int }_{-\infty }^{0}{\mathscr{N}}(\mu ,{\sigma }_{150})d\mu $$
2. Expected trip-over numbers in a two-year period: We can calculate expected yearly trip numbers per age using the equation: tripping−overs per year = *p*_*trip*_·stair climbs per year. As there were no population-based yearly stair climb counts available, we adapted data from a study conducted by Coupland and colleagues, which showed that people in their 50s in England climbed 10 flights of stairs daily on average^49^. We then normalised stair climbing counts according to activity levels (i.e. stair climbing hours per day) acquired from Health Survey for England 2008 by 10 year age groups (16–75+ years)^50^.

We then compared model prediction with two-year falls on stairs statistics from a cohort study by Talbot and colleagues^51^.

## Results

### Experiment 1: Sensorimotor variability of the FOHEPO task

**Figure 3:**
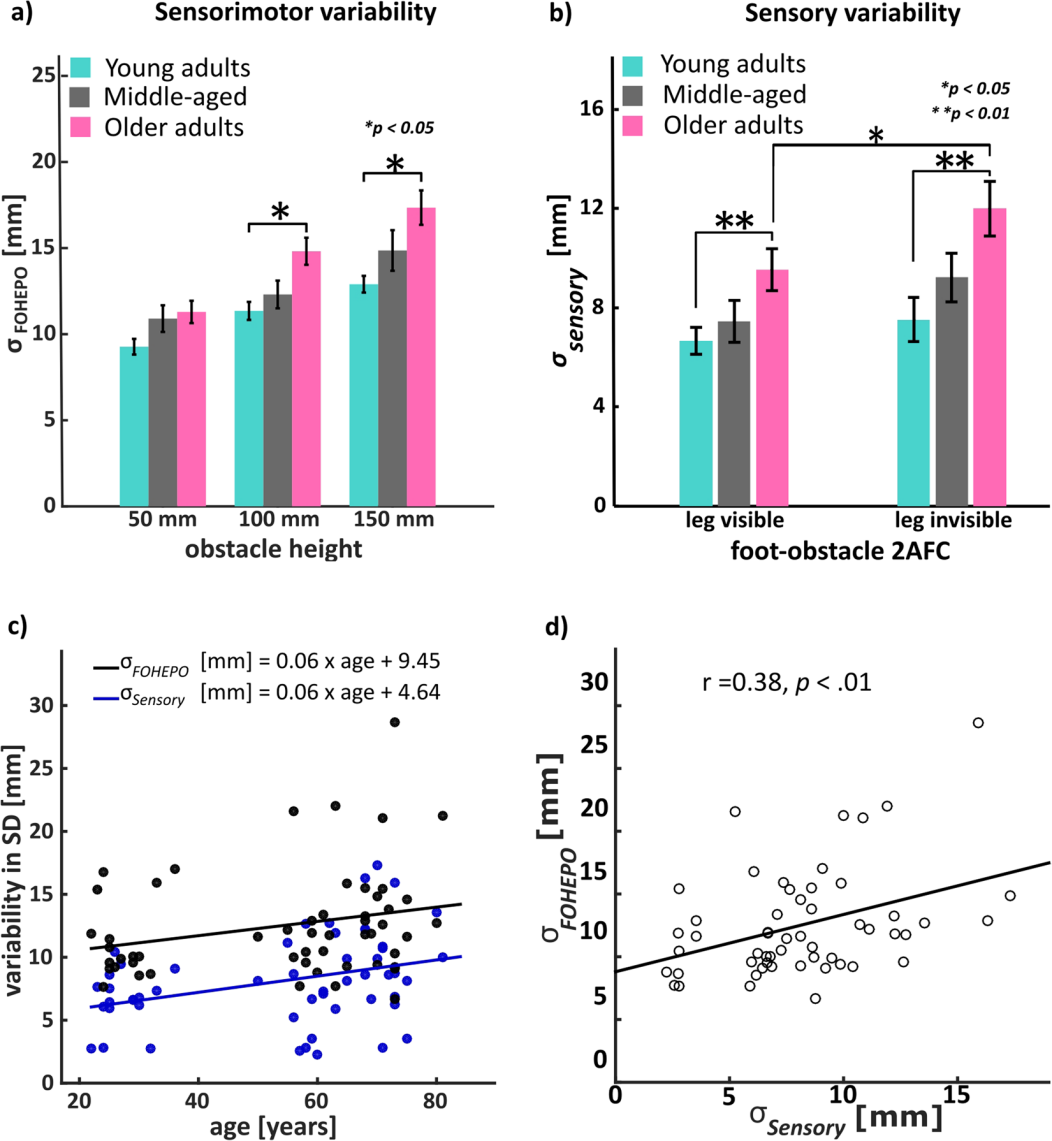
Sensorimotor and sensory variability under different experimental conditions. **(a)** Group means of the sensorimotor variability of the FOHEPO task of each height condition. Significant age group differences were found in 100 mm (*p* = 0.012) and 150 mm (*p* = 0.014) conditions. **(b)** Group means of sensory variability in the foot-obstacle 2AFC, leg-visible and -invisible conditions. Older subjects had higher levels of sensory variability than the young subjects in both leg-visible (*p* = 0.032) and leg-invisible (*p* = 0.015) conditions. Bonferroni t-test showed the sensory variability of older people in leg-invisible condition increased significantly compared to leg-visible condition (*p* = 0.02). **(c)** Scatter plot of age and both sensorimotor and sensory variability in the 100 mm condition as a linear function of age. Age was a significant, positive predictor of both sensorimotor (*r* = 0.30, *p* = 0.026) and sensory variability (*r* = 0.35, *p* < 0.01). Across adulthood, sensorimotor and sensory variability both increased at 0.06 mm per year averagely. Similar graphics showing the sensorimotor variability of the 50 mm and 150 mm conditions versus age can be found in the Supplementary Material Fig. 2. (**d**) There was also a significant positive correlation between sensorimotor and sensory variability (*r* = 0.38, *p* = 0.004). The results of **(c)** and **(d)** imply that the sensory noise being one cause underlying more variable movement in ageing.

We designed and applied the FOot HEight POsitioning (FOHEPO) task to measure end-point variability during movements that were biomechanically comparable to foot clearance when stepping to elevated surfaces. We used standard deviations to represent the sensorimotor variability of the FOHEPO task because Kolmogorov-Smirnov Goodness-of-Fit tests confirmed the normality of the foot height distributions (Supplementary Material Fig. 1). The mean sensorimotor variability measured for each group of each condition can be seen in Table 1. Sensorimotor variability increased significantly with age, as revealed by the age main effect (*F*_(2,70)_ = 4.71; *p* = 0.012) of Age × Height × Foot ANOVAs. Post-hoc analysis displayed a significant difference between the older and young (*p* = 0.003), but not the middle-aged and young (*p* = 0.63). A significant height effect was also found (*F*_(2,140)_ = 55.13; *p* < 0.001). Further, because of a marginally significant age-group/obstacle height interaction (*F*_(4,140)_ = 2.04, *p* = 0.09), we analysed the simple effect of age on each height condition using Age × Foot ANOVAs. Older people performed significantly more variably than young when matching obstacles at 100 mm (*p* = 0.012) and 150 mm (*p* = 0.014) (see Fig. 3a).

**Table 1:** Sensorimotor variability of the FOHEPO task by age groups, feet and heights.

	Young (M±S.D)	Middle-aged (M±S.D)	Older (M±S.D)
**L50**	10.0 (4.0)	10.5 (4.4)	11.2 (4.3)
**R50**	8.6 (2.7)	11.3 (5.0)	11.3 (5.2)
**L100**	12.0 (4.2)	12.1 (4.2)	14.8 (5.9)
**R100**	10.7 (3.7)	12.6 (5.5)	14.8 (5.5)
**L150**	13.6 (3.5)	15.3 (7.5)	17.5 (7.2)
**R150**	12.2 (3.8)	14.4 (6.8)	17.2 (7.3)

Expressed in mean (S.D) unit: mm.

A multiple linear regression was carried out to investigate whether age, foot and obstacle height together could significantly predict participants’ sensorimotor variability. The results of the regression indicated that the model explained 19.1% of the variance (*R*^2^ = 0.19) and the model was a moderately significant predictor of sensorimotor variability (*F*_(3,434)_ = 34.24, *p* < 0.001; multiple correlation coefficient *R* = 0.44). While age (*β* = 0.074, *p* < 0.001) and obstacle height (*β* = 0.074, *p* < 0.001) contributed significantly to the model, foot did not (*β* = −0.528, *p* = 0.27). A test of quadratic trend was statistically non-significant (*p* = 0.20). Having found that the FOHEPO task, as in previous studies, showed increased sensorimotor variability with age. We now proceed to focus on sensory components within this context.

### Experiment 2 and 3: Sensory variability in the foot-obstacle 2AFC

To quantify sensory variability, we used foot-object height discrimination psychophysics. Sensory variability (Fig. 3b and Table 2) showed significant age group (*F*_(2,52)_ = 6.07, *p* = 0.004) and condition effects (condition mean ± SE: leg-visible = 7.8 ± 48 mm, leg-invisible = 9.5 ± 60 mm; *F*_(1,52)_ = 8.40, *p* = 0.005), but no interaction. Pair-wise comparison found that older people had higher levels of sensory variability than the young (*p* = 0.004). Importantly, in post-hoc analysis, the condition effect was only significant in the older group (*p* = 0.02), but not in the young (*p* = 0.39) and middle-aged (*p* = 0.11), which implied that absent visual information of foot height negatively impacted older people’s performance more than the other two groups.

**Table 2:** Sensory variability by age groups and experimental conditions/sensory modalities.

	Young (M ± S.D)	Middle-aged (M ± S.D)	Older (M ± S.D)
**Foot-obstacle 2AFC-LV**	6.7 (2.2)	7.4 (3.5)	9.5 (4.2)
**Foot-obstacle 2AFC-LI**	7.5 (3.7)	9.2 (4.1)	12.0 (5.1)
**Proprioceptive noise**	6.7 (3.6)	7.9 (4.3)	10.5 (4.9)
**Visual noise of obstacle**	5.2 (2.4)	6.6 (3.2)	6.5 (3.0)

Expressed in mean (S.D) unit: mm.

We performed a linear regression on sensory variability against age. As can be seen from Fig. 3c (blue dots and line), age was a significant predictor of sensory variability. The regression equation was sensory variability (mm) = 4.64 (mm) + 0.06 · age (*r* = 0.35, *p* < 0.01). There was also a positive correlation between sensorimotor and sensory variability (Fig. 3d.; *r* = 0.38, *p* = 0.004). A partial correlation between an individual’s sensorimotor variability at 100 mm and age whilst controlling the effect of sensory variability was then conducted. We found no positive partial correlation between sensorimotor variability and age whilst taking account the mediating effect of sensory variability (*ρ* = 0.14, *p* = 0.31). This result indicates that sensory variability had a significant influence on ageing-related sensorimotor variability increases. Having shown that sensory variability increased with age, we then applied sensory psychophysics of object height discrimination and used the computational rules of sensory cue integration to estimate the variability of proprioception and vision separately.

### Assessing the variability of individual sensory modalities

#### Proprioceptive variability of the foot estimates

**Figure 4:**
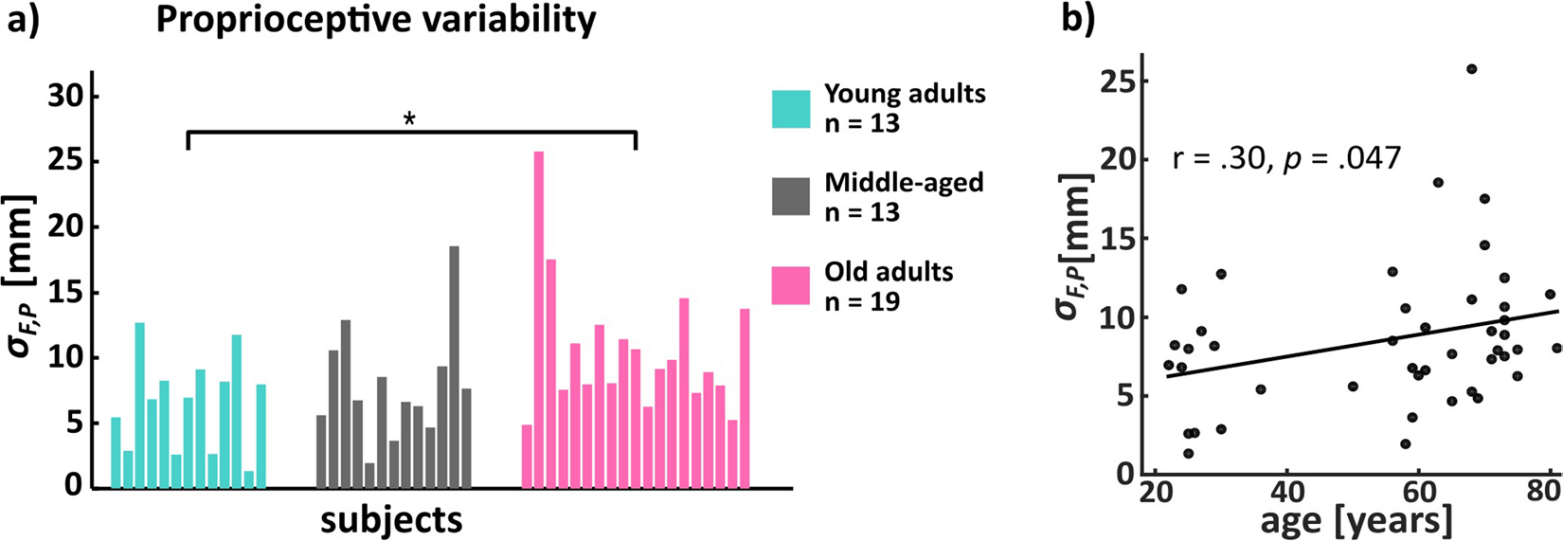
Proprioceptive variability. **(a)** There was a significant age group effect (*F*_(2,44)_; *p* = 0.048). Pairwise comparison showed that older adults had higher proprioceptive variability compared to young (Bonferroni t-test *p* = 0.055). Each bar represents a single subject. It should be noted that the sampling numbers were different from those of sensory experiments. Some participants’ data (4 out of 17 young adults, 4 out of 17 middle-aged and 2 out of 21 older adults) had to be excluded because negative values of ${\sigma }_{F,P}^{2}$ were obtained when using eqs (3) and (4) to compute proprioceptive variability. **(b)** Scatter plot of age and proprioceptive variability. Age was a positive predictor of proprioceptive variability (*r* = 0.30; *p* = 0.047).

As can be seen from Fig. 4a, there was a significant age group effect (*F*_(2,44)_; *p* = 0.048). Pairwise comparison showed that older adults had higher proprioceptive variability compared to young (*p* = 0.055). The middle-aged group did not have significant differences to any of the other two groups. A linear regression on proprioceptive variability against age showed a significant positive correlation between proprioceptive variability and age (Fig. 4b; *r* = 0.30; *p* = 0.048). There was also a significant positive correlation between sensory and proprioceptive variability (Supplementary Material Fig. 3a; *r* = 0.53; *p* < 0.001). Importantly, the result of a partial correlation between sensory variability and age whilst controlling the effect of proprioceptive variability was not significant (*ρ* = 0.20, *p* = 0.19), suggesting that the ageing-related increase of sensory variability was likely due to the increase proprioceptive variability. It should be noted that we excluded individuals’ data (4 out of 17 in young, 4 out of 17 in middle-aged and 2 out of 21 in older) from statistical analysis because negative values of ${\sigma }_{F,P}^{2}$ were obtained when using Eqs (3) and (4) to compute proprioceptive variability.

#### Visual variability of the obstacle and foot estimates

**Figure 5:**
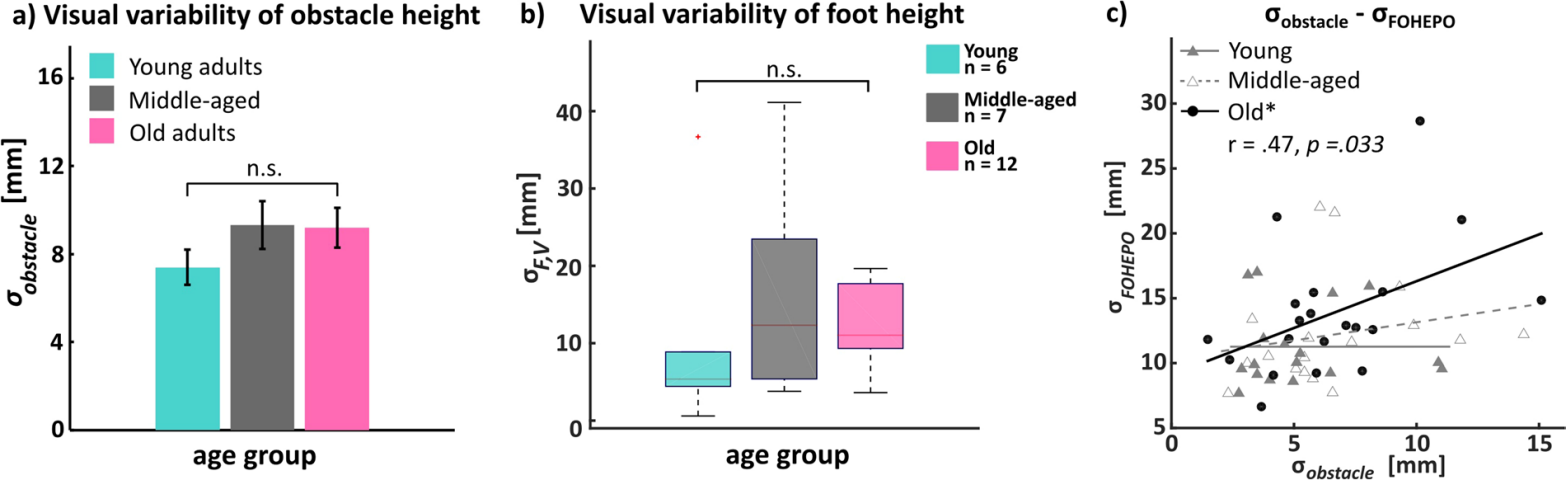
Visual variability of obstacle and foot height **(a)** Mean visual variability of obstacle height *σ*_*obstacle*_ of each age group. There was no significant age group differences by ANOVA. Error bars indicate SEM. **(b)** Visual variability of foot height *σ*_*F*,*V*_. Individual visual variability was acquired by using Eq. (2), which assumed people follow the Bayesian integration rule when combining visual and proprioceptive information to estimate their foot heights. There was no significant age group effect. **(c)** Scatter plot of visual variability of obstacle height and sensorimotor variability. Only in the older people group, there was a significant positive correlation between visual variability and sensorimotor variability (*r* = 0.47; *p* = 0.033). In the young and middle-aged groups, no such relationship was observed.

The visual variability of obstacle height had neither age group difference (*F*_(2,58)_ = 1.3; *p* = 0.28) (Fig. 5a) nor significant correlation (*p* = 0.20) with participants’ age. We performed a BF analysis to quantify how much more likely the non-significant results were to occur if the null hypothesis is true, compared to if the alternative hypothesis is true. BF between the older and young was 1.80 and between the older and middle-aged was 4.25, supporting the null (i.e. no difference) over the alternative hypothesis. Additionally, the result of a partial correlation between sensory variability and age whilst controlling the effect of visual variability remained to be significant (*ρ* = 0.32, *p* = 0.033), indicating that visual variability had little influence in controlling for the relationship between sensory variability and age.

However, we also found that only in the older group, there were significant positive correlations between the visual variability of obstacle height and sensorimotor variability (Fig. 5c; *r* = 0.47; *p* = 0.033) as well as sensory variability (*r* = 0.49; *p* = 0.023, see Supplementary Material Fig. 3b). This implied that individual differences of visual precision affect older people’s sensorimotor precision more than the other two groups.

Based on the assumption of Bayesian integration rule^11,38^, we attempted to calculate the visual variability of foot estimates using Eq. (2). While negative values of ${\sigma }_{F,V}^{2}$ were found in a notable proportion of participants. we excluded those cases. In discussion, we examined the possible reasons behind the detection of negative values. The medians of ${\sigma }_{F,V}^{2}$ was found to be 5.4 mm in the young (N = 6), 12.3 mm in the middle-aged (N = 7) and 11.0 mm in the older (N = 12) (Fig. 5b). Although Kruskal-Wallis H test was not significant (*p* = 0.24), the result needs to be interpreted with extra-caution because many subjects’ data were excluded.

### Modelling tripping-over occurrence by age

**Figure 6:**
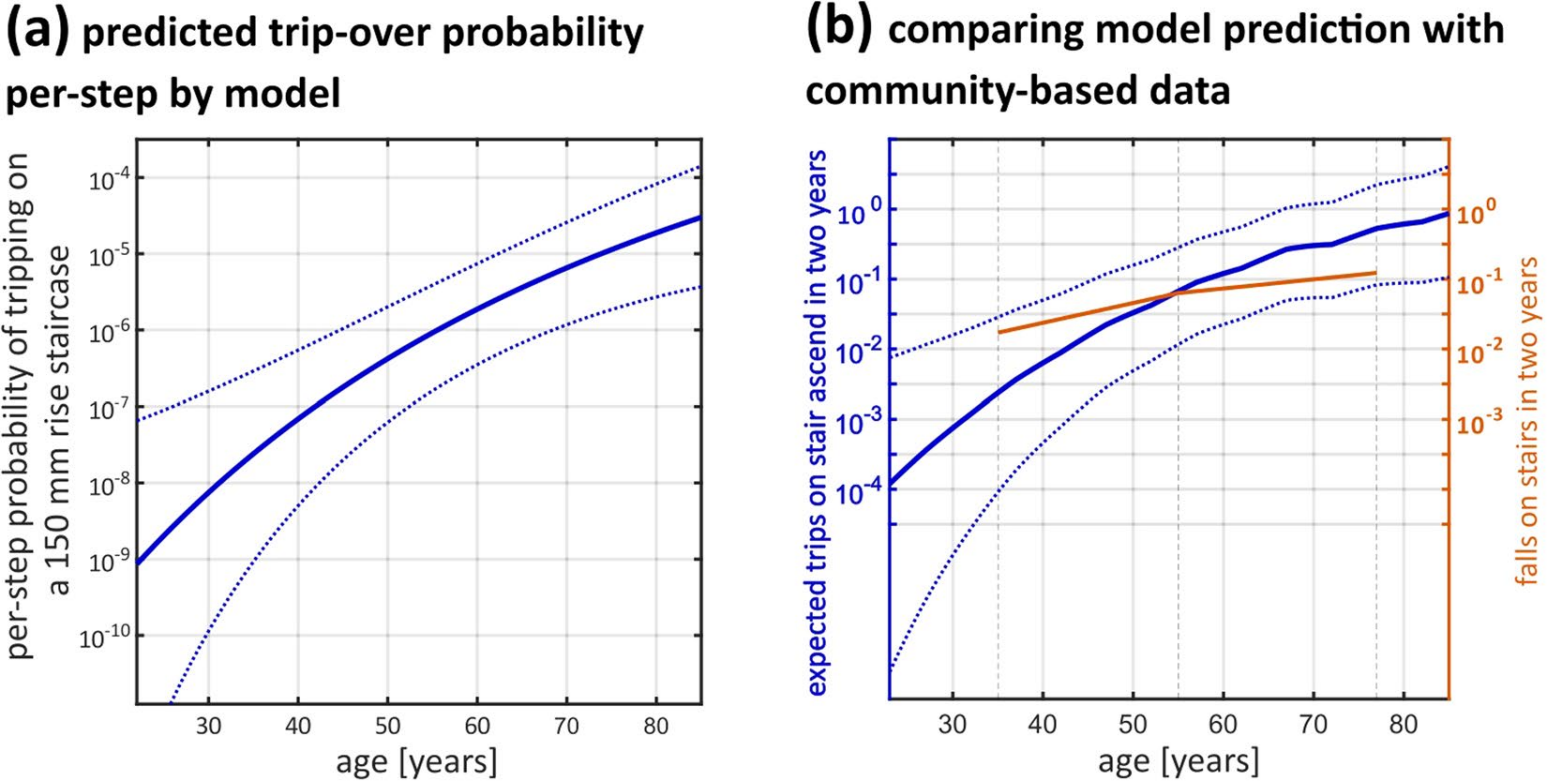
**(a)** We modelled the tripping probability when healthy adults aged 22–84 years step onto a 150 mm rise staircase as a function of age. The model was based on our result of FOHEPO task sensorimotor variability as a linear function of age. The per-step tripping probability increased by more than 10^4^ times from 20+ to 80+ years. Dashed lines represent the 95% confidence intervals. **(b)** Compare model prediction to reported data. The blue line represents expected trip-over number during a period of two years as a function of age. The predicted trip-over count was calculated by multiplying the tripping probability shown in **(a)** with stair-climbing counts normalised to age according to previous research^49,50^. As can be seen, the (log) expected trip-over occurrence increased nearly monotonically, from 10^−4^ at 22 years to about 1 at 84 years in a two year period. Dashed lines represent the 95% confidence intervals of model prediction. The orange line represents community-based fall numbers of three age groups: young (mean 35 years), middle-aged (mean 55 years) and older (mean 77 years) people, reported in a two-year retrospective study by Talbot and colleagues^51^. As can be seen, the model prediction trend was broadly similar to community-based data.

As can be seen from Fig. 6b, expected tripping-over numbers over a period of 2 years increased from 10^−4^ to 1. This progressive increase of predicted trips with age was generally consistent with the fall-upon-stair data from a population study^51^. However, the extent of reported fall increase was not as drastic as prediction. From aged 30s to 70s, falls on the stairs increased around 10-fold. Logistic regression showed age, but not sensorimotor variability per se, a significant predictor of fall history in our participants (Supplementary Material Table 2).

## Discussion

Greater gait variability, specifically, greater MFC variability, has been identified as an important marker of increased fall risk in older people. How the changes of the sensorimotor system in ageing, especially alterations in individual sensory systems, are related to increased gait variability in older people has mostly been unexplored. Here, we demonstrate a link between sensorimotor variability and MFC variability increases in older people. We further show that the increased sensory variability is a crucial factor in the age-related increases of sensorimotor variability. We then specifically quantified the variability of individual sensory modalities, i.e. proprioception and vision, and characterised their relationship to age-related increases of sensory variability. The key finding is that proprioceptive but not visual variability correlated with the age-related increases of sensory and sensorimotor variability. We additionally predicted fall probability by age based on the linear relation between our sensorimotor variability results and age.

The two common ageing motor features are increased end-point variability and slower movements^16–22^. In this study, we focused on end-point variability and provided sufficient time (5 seconds to observe and prepare and 4 seconds to move) for task completion. The results showed that older participants completed equal amount of trials (older 99.4 ± 1.4%; young 99.4 ± 1.0%) but spent more time on foot matching (1.91 seconds in older people 1.69 seconds in the young), compared to the young (Supplementary Material Table 1). Besides, no speed-accuracy correlation was shown (Supplementary Material Fig. 5). It is therefore rational to conclude that our observation of increased sensorimotor variability with age did not result from older people trading precision with speed. This is important because older adults typically display higher gait variability, independent of slower walking speed, in gait biomechanics^52,53^.

We used regression to examine sensorimotor variability changes across adulthood and found an increase roughly by 0.07 mm per year. This trend appears to be significantly linear and not characterised by sudden jumps. In the small number of studies investigating variability in perception-action tasks across adult lifespan, some studies identified, similar to our finding, that variability increases as early as the age of 50^54,55^ while some found that variability remains stable until age 60^56,57^. The discrepancies between studies^56,57^ could have been caused by differences in task nature. The FOHEPO task needs collaboration of multiple sites in the sensorimotor system, which show diverse trajectories along the course of ageing^19^. For example, studies have found lower motor neurons showing no evidence of loss before the age 60^58,59^ and upper motor neurons in the brain continuously decreasing across adulthood^60^. The distributed computations needed for the FOHEPO task may be the reason that the performance degraded steadily across age. This monotonous degradation is a potential candidate for early risk detection while the pathologies causing it are prospective targets of activity modifications to prevent later impairments. However, the linear regression model explained only about 20% variance (*R*^2^ = 0.19), indicating significant amounts of sensorimotor variability were influenced by factors other than age. Investigations into additional factors related to sensorimotor variability are needed before accurate risk evaluation can be reached.

Sensorimotor variability is composed of sensory variability, motor variability and internal mechanisms mediating the two^61^. We find that sensory variability, measured via experiment 2, was significantly higher (by 42%, 2.8 mm) in the older than the young. The quantitative increase of sensory variability matched which in sensorimotor variability (by 30%, 3.4 mm). When controlling for the effect of sensory variability by partial correlation, the positive correlation between sensorimotor variability and age was explained away. We concluded that sensory variability is the primary driver for age-related increases in sensorimotor variability. We further estimated the three primary factors constituting sensory variability in the task, proprioceptive variability in estimating the own foot heights, visual variability in estimating the own foot heights and visual variability in estimating the obstacle height. We demonstrated that proprioceptive variability increased significantly by 56% in the older group. Additionally, the ageing-related increases of sensory variability were explained away by proprioceptive but not visual variability, as demonstrated by the results of partial correlations. This provides evidence supporting the importance of proprioception to gait control. Previous studies revealed that impaired proprioception during ageing impacts stance and balance control^62,63^ with a focus on static scenarios. We were able to show here that increased proprioceptive variability critically affects sensorimotor variability and as a result, the variability with which an ageing person clears over an obstacle (foot clearance variability).

We hypothesised that participants integrate information from both vision and proprioception to boost foot position estimate precision. This would result in a decrease of sensory variability when participants were allowed to use visual information of foot positions, as shown in the results. Importantly, the degree of precision enhanced by visual information differed greatly between age groups. Older people benefited substantially from vision (sensory variability reduced from 12.0 to 9.5 mm with a post-hoc t-test *p* = 0.02), while young and middle-age did not show significant differences. The result is compatible with previous studies showing enhanced benefits from multisensory integration in older people compared to the young^64^. It is also in agreement with research discovering that older people compared to the young rely more on vision to maintain balance^65^. This poses a general question, why sensory precision did not improve notably in the young and middle-age when they integrated the visual information of foot estimates. This observation can be explained by the Bayesian integration rule^11,38^, which states that the weights of individual sensory modalities are inversely proportional to their variance. Suppose one sensory modality has overwhelmingly lower variability than the other, weighting would exceedingly favour the modality with lower variability and post-integrated sensory variability would be nearly identical to the smaller mono-sensory variability. Previous research has shown that young subjects mostly reply on proprioception in hand depth judgment, as proprioception is more precise than vision in the depth dimension (i.e. the radial direction with respect to observers)^39^. The optical geometry of foot height estimation is the same as those of hand depth estimation. Had it been a similar but more extreme case in the difference between proprioceptive and visual precision, subjects would have depended much on proprioception and shown a post-integrated sensory variability almost indistinguishable from proprioceptive one. This is what we identified in our young and middle-aged participants. On the contrary, older people increased their reliance on vision based on the Bayesian integration rule to react to raised proprioception variability. By doing so, they significantly enhanced their performance.

An integration policy strongly replies on more precise proprioception in the young and middle-aged also provides an explanation why a non-trivial number of negative values were acquired when applying eqs (3) and (4) to recover the foot height estimate visual variability. In this circumstance, the reduction of variance resulting from sensory integration becomes so small that the measurement variability could have exceeded the effect and caused ${\sigma }_{F,P}^{2}$ smaller than $\frac{{\sigma }_{F,V}^{2}\cdot {\sigma }_{F,P}^{2}}{{\sigma }_{F,V}^{2}+{\sigma }_{F,P}^{2}}$. Indeed, we found the mean proprioceptive variability of those have negative values obtained in calculation was 5.8 mm, much lower than the mean of those positive values obtained (mean 9.6 mm; *p* = 0.01). The small number of foot visual variability estimates did make it difficult to draw a conclusion from the remaining data even though our result was parallel to the result of obstacle visual variability.

Still, an absence of raised obstacle visual variability in our older participants was surprising considering that visual problems are thought a prominent characteristic during ageing. One reason could be that the primary source of increased visual variability during ageing is increased light scatters due to clouding lenses, i.e. cataract^66^. We reviewed our older participants’ health status questionnaires. They had not only corrected-normal sight but also a very low prevalence of cataracts (2 out of 26 in older and 1 out of 18 in middle-aged group compared to 40% prevalence in population-based studies^67,68^). This could have been the reason that visual variability in the sampling older population was equivalent to the young. However, while showing no inter-group difference, the inter-individual differences of visual variability was a positive predictor of increased sensorimotor variability in older people. The finding can be recognised as supporting evidence of increased reliance on visual information with age. It also identifies the danger of over-reliance on a single sensory domain for compensation and raises the importance of physical training of proprioceptive capabilities, which has been shown to promote retention^69^ and improvements^70^ on proprioception.

Developing an effective predictive behavioural marker for fall risk screening is important but challenging. The benefits of using the FOHEPO and related sensory tasks to evaluate fall risk are repeatable during follow-ups and capable of recognising specific sensory modality changes to set up rehabilitation plans. We attempted to link sensorimotor variability to tripping risk by building up an inference module based on the linear model between sensorimotor variability and age. Though the trend between our prediction and the surveillance data were similar, the result should be treated with extra caution. First, as has been mentioned, the linear correlation between sensorimotor variability and age was not strong. Second, additional logistic regression (Supplementary Table 2) showed that in our sampling population, age but not individual sensorimotor variability predicted whether participants experienced falls in the past two years. Taking these findings together, we acknowledge sensorimotor variability and falling in daily life can be influenced by multiple factors that we cannot exhaustively examine in a single study. We expect future work to elucidate other elements interacting with sensorimotor variability values and falls in older people.

To conclude, the current study, to the knowledge of the authors, is the first experimental evidence to show a connection between increased sensorimotor variability and increased foot clearance variability. Importantly, we systematically examined the relationship between changes in individual sensory modalities, overall sensory variability and sensorimotor variability. We demonstrate that the experimental protocols can pinpoint a specific deficit of the sensory systems, namely increased proprioceptive variability, critically linking to the increased sensorimotor variability of the tested ageing subjects. The finding provides the neural control understanding of falls and also has practical implications for the future development of risk screening tools and targeted rehabilitation exercise.

## Electronic supplementary material

Supplementary Information
