# Supplementary material for: Decomposing sensorimotor variability changes in ageing and their connection to falls in older people
Source: Sci Rep. 2018 Sep 28;8:14546. doi: 10.1038/s41598-018-32648-z (PMC6162289; doi:10.1038/s41598-018-32648-z)
Supplement: Supplementary file 1 — Supplementary Information [file 41598_2018_32648_MOESM1_ESM.pdf]

Decomposing sensorimotor variability changes in ageing and their connection to falls in older people

Chin-Hsuan Lin; A Aldo Faisal

Supplementary Material

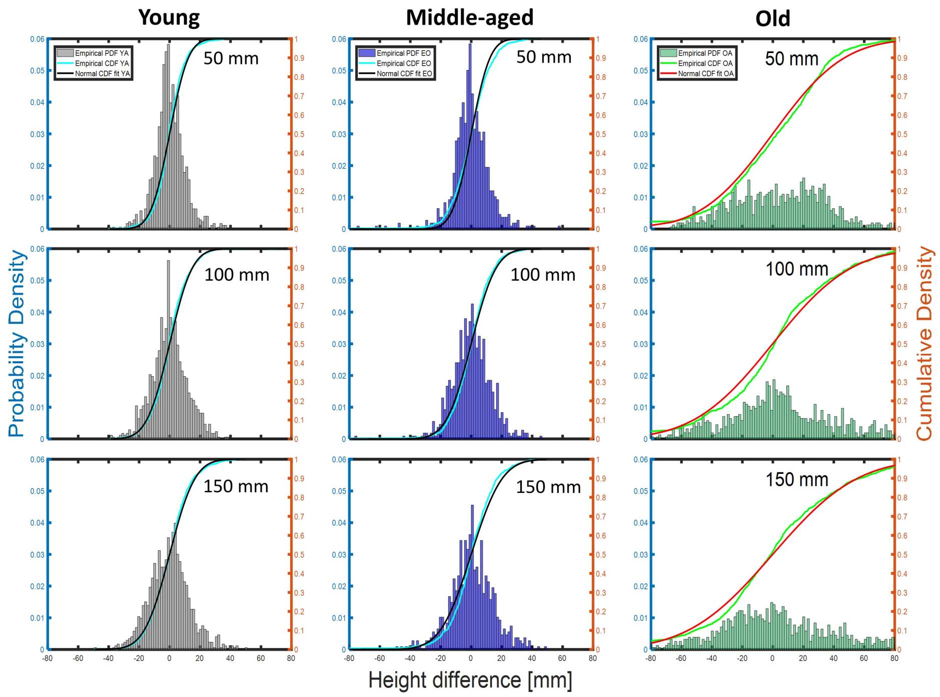

Supplementary Fig. 1: We calculated the difference between foot height and individual mean foot height of each condition on a trial-by-trial basis and thus obtained distributions of height differences (differences to each participants mean in the same condition). The probability density function (PDF), empirical and fitted cumulative density functions of difference foot placement in the young (N = 29, left column, grey), middle-aged (N = 18, middle column, blue) and old adult (N = 26, right column, green) groups are presented. Data of each height condition is presented in individual diagram. Each individual contributed 40 trials to each diagram. The empirical cumulative density functions (CDFs) (cyan lines for the young and middle-aged, green lines for the Old) are highly corresponding to fitted Gaussian distributions (black for the young and middle-aged, red lines for the Old). Kolmogorov-Smirnov Goodness-of-Fit tests also supported that the distributions of foot placement fit Gaussian.

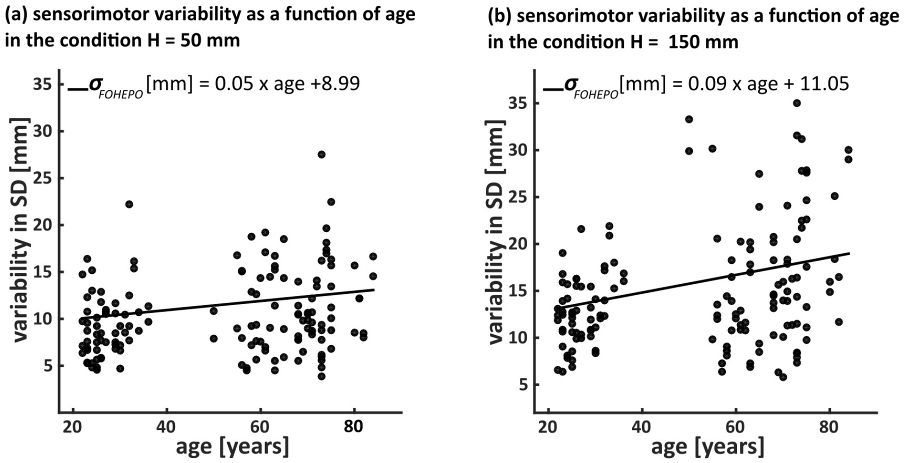

Supplementary Fig. 2: Scatter plot of age and sensorimotor variability in the **(a)** 50 mm and **(b)** 150 mm conditions as a linear function of age. Age was a significant, positive predictor of both conditions ($r = .25$, $p = .002$ and $r = 0.32$, $p < .001$ respectively). On average, sensorimotor variability increased at 0.05 mm in the 50 mm and 0.09 mm in the 150 mm conditions per year respectively.

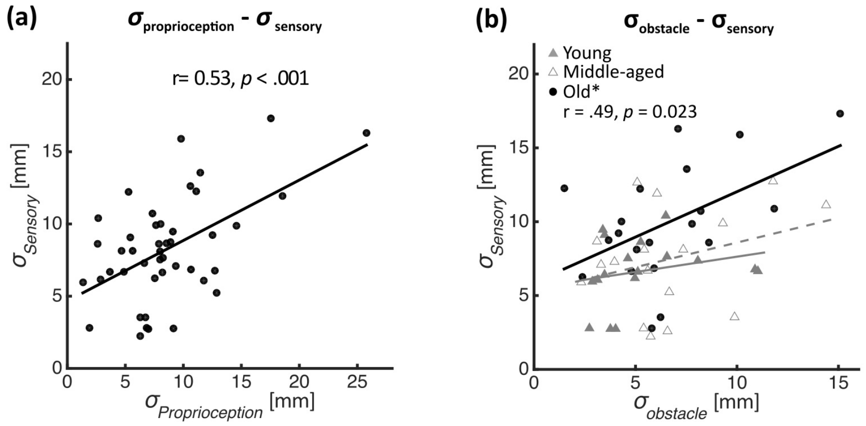

Supplementary Fig. 3: **(a)** Scatter plot of sensory and proprioceptive variability. Proprioceptive variability was a positive predictor of sensory variability ($r = .53$; $p < .001$). **(b)** Scatter plot of visual variability of obstacle height and sensory variability. Only in the elderly group, there was a significant positive correlation between visual variability and sensorimotor variability $r = .49$; $p = .023$. Neither in young or middle-aged groups, such a relationship was observed. This relationship was similar to the relationship between visual variability of obstacle height and sensorimotor variability, as can be seen in Fig. 5.c. in the manuscript.

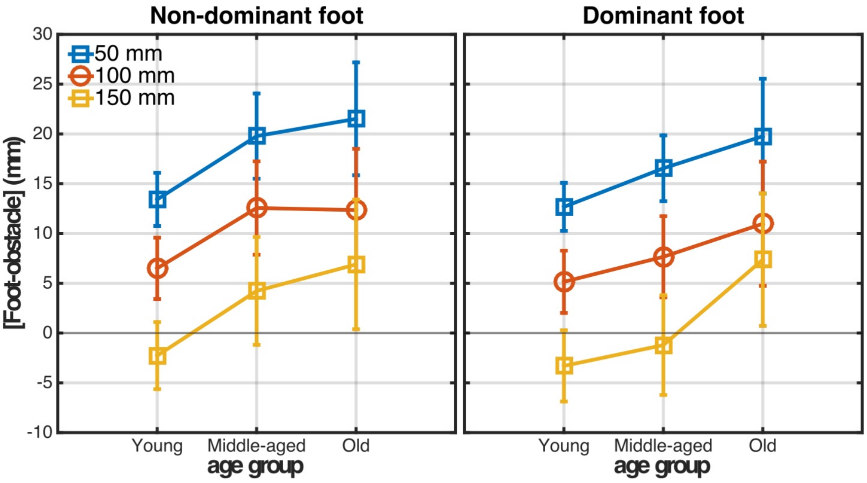

Supplementary Fig. 4: This figure showed the mean of obstacle height subtracting from foot height (i.e. difference height or bias of foot placement) of each group for each condition. Although all participants systemically placed their feet higher than the height of the obstacle (grand mean ± 95% confidence intervals: 9.5 ± 4.2 mm), the trend diminished with increased obstacle height, with a three-way mixed design ANOVA showing a significant height effect ($F_{(2,69)} = 62.68$, $p = .001$). Moreover, there was no statistical significance of age-group ($F_{(2,70)} = 0.87$, $p = .42$), although the biased foot placement was slightly more apparent in the elderly than the young and middle-aged. Error bars represent SEM.

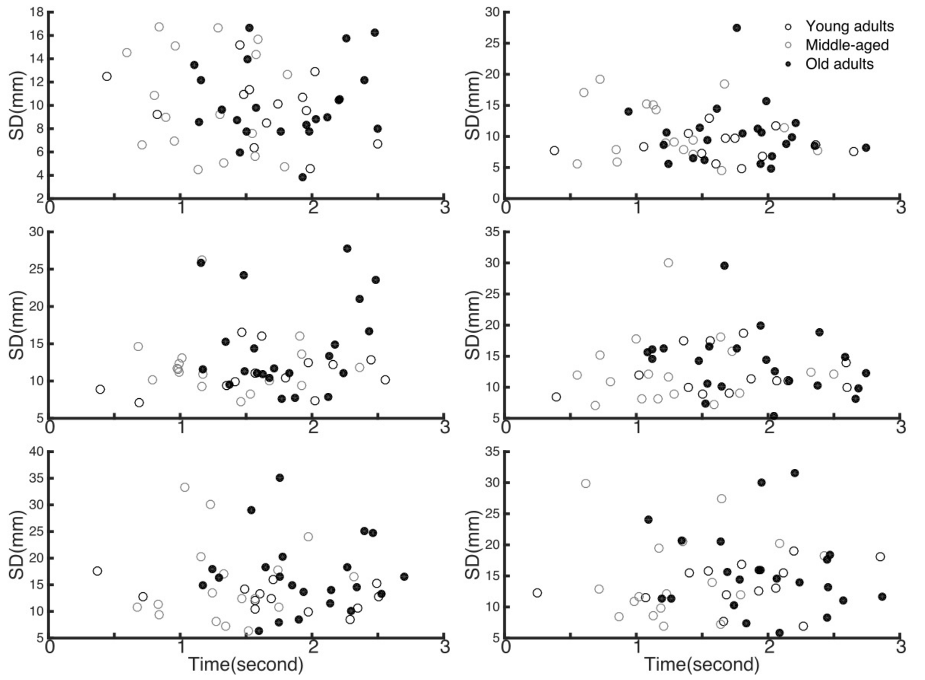

Supplementary Fig. 5: Scatter plots of mean trial time and sensorimotor variability in the 50 mm (upper two) and 100 mm (middle two) and 150 mm (lower two) conditions. In all three groups and all height conditions, no correlation between trial time and sensorimotor variability was found. This indicates that the participants, especially the older subjects who had the longest trial time, did not try to achieve high precision by lowering their speed (i.e. no speed-precision trade off).

Supplementary Table 1: The left column showed the percentage of trials completed All three groups completed more than 99% of trials. There was no statistical difference in completed trial number. The right column showed trial time (mean ± 95% confidence intervals) of three age groups. Old adults spent significantly longer in a trial compared to the young and middle aged adults.

	**trial completed(%)**	**trial time mean± 95% CI (s)**
**Young**	99.4(1.0)	1.69(0.21)
**Middle-aged**	99.8(0.6)	1.31(0.23)
**Old**	99.4(1.4)	1.91(0.20)

Supplementary Table 2: Logistic regression analysis of 73 participants' fall history showing that age but not individual sensorimotor variability a significant predictor whether participants fell in the past two years.

Predictor	β	S.E.β	Wald	df	Sig.	odds ratio
Variability	-.007	.058	.014	1	.904	.993
Age	.087	.036	5.787	1	.016	1.091
Constant	-7.048	2.431	8.405	1	.004	NA
Test			Chi-Square	df	Sig.	
Goodness-of-fit test						
Hosmer & Lemeshow			4.830	8	.776	

Note: Cox & Snell R Square = .167 Nagelkerke R Square = .292. NA: not applicable
